# ﻿*Bambusarushunii* (Poaceae, Bambusoideae, Bambuseae), a new bamboo species from Guangdong, China

**DOI:** 10.3897/phytokeys.253.143389

**Published:** 2025-03-04

**Authors:** Jing-Bo Ni, Meng-Ling Li, Shu-Peng Dong, Yi-Hua Tong

**Affiliations:** 1 State Key Laboratory of Plant Diversity and Specialty Crops, Key Laboratory of National Forestry and Grassland Administration on Plant Conservation and Utilization in Southern China, South China Botanical Garden, Chinese Academy of Sciences, Guangzhou, Guangdong 510650, China; 2 South China National Botanical Garden, Guangzhou 510650, China; 3 Laboratory of Plant Resources Conservation and Sustainable Utilization, South China Botanical Garden, Chinese Academy of Sciences, Guangzhou 510650, China

**Keywords:** *Bambusa*, Guangdong, morphology, woody bamboo

## Abstract

A new bamboo species, *Bambusarushunii*, from Yangjiang City, Guangdong Province, China, is described and illustrated in this paper. The new species resembles *B.gibba* and *B.dissimulator* in having branchlets specialised into weak thorns at the lower nodes of culms, but can be easily distinguished from the latter two by having one or two extremely shortened internodes at the culm base, glabrous internode, culm leaf sheath being dark brown strigose on the central part and with a nearly truncate or slightly obliquely truncate apex, relatively high culm leaf ligule, culm leaf blade base not narrowed, extending outwards and contiguous with auricles and the glabrous foliage leaf with a ciliate ligule margin.

## ﻿Introduction

Bamboos, belonging to the subfamily Bambusoideae of Poaceae, are of great ecological, social and economic value as building material, household utensils, vegetables, raw material for making paper and musical instruments (Zhang et al. 2012; Ahmad et al. 2023). There are nearly 1700 species in 136 genera of bamboos worldwide, classified into three tribes, tropical Bambuseae Kunth ex Dumort., temperate Arundinarieae Asch & Graebn and herbaceous Olyreae Kunth ex Spenn (Sungkaew et al. 2009; Kelchner and Bamboo Phylogeny Group 2013; Clark and de Oliveira 2018; Soreng et al. 2022).

The genus *Bambusa* Schreber is the most widely cultivated woody bamboo genus with more than 150 species, of which about 80 species are distributed in southern and south-western China (Xia et al. 2006; Vorontsova et al. 2016; Clark and de Oliveira 2018). It forms the BDG complex (*Bambusa*-*Dendrocalamus*-*Gigantochloa* complex), which is considered to be the “core Bambusinae” by Goh et al. (2013), together with *Dendrocalamus* Nees, *Gigantochloa* Kurz ex Munro and other closely-related genera (Zhou et al. 2017; Liu et al. 2020). The paraphyly of *Bambusa* was confirmed by much phylogenetic research based on plastome and nuclear DNA data (Yang et al. 2010; Zhou et al. 2017; Liu et al. 2020). Moreover, none of the four subgenera within *Bambusa* was supported as monophyletic (Yang et al. 2010; Liu et al. 2020). Although there are intractable phylogenetical problems due to a history of rapid diversification and putative introgression events, it is now generally believed that some floral characters, such as morphology of rachillas, lodicules and filaments can be used to distinguish *Bambusa*, *Dendrocalamus* and *Gigantochloa*, which also possess phylogenetic signals (Loh et al. 2000; Goh et al. 2010; Liu et al. 2020). Specifically, disarticulated rachillas, 2–3 lodicules and free filaments could serve as suitable diagnostic characters to distinguish *Bambusa* from the other two genera (Wong 1995; Chia et al. 1996; Xia et al. 2006; Qin et al. 2022).

Guangdong, with more than 60 *Bambusa* species, is one of the provinces that harbour the highest biodiversity of *Bambusa* in China (Xia and Lin 2009). It belongs to the East Asian monsoon region, with subtropical and tropical climates from north to south, respectively. The annual average temperature in Guangdong Province is 18–22 °C and the rainfall in Guangdong Province is mainly concentrated from April to September, with an average precipitation from 1500 mm to 2000 mm each year. Thus, the hydrothermal condition of Guangdong is very suitable for the growth of *Bambusa* species. During several field investigations in Yangchun County, Yangjiang City, Guangdong Province, we found three populations of an unknown species of *Bambusa*. This species bears extremely shortened internodes at the basal culm, which is very rare in *Bambusa*. After comparison with morphologically similar species, it is concluded that this unknown species has not been described before. Therefore, it is described as a new species in this paper.

## ﻿Materials and methods

Specimens of this new species were collected during two field surveys to Dongping Town, Yangdong District, Yangjiang City, Guangdong Province in 2016 and Tanshui Town, Yangchun County, Yangjiang City, Guangdong Province in 2023. Voucher specimens were deposited in the Herbarium of the South China Botanical Garden (IBSC), Chinese Academy of Sciences. Flowering materials were dissected under a stereomicroscope (Mshot-MZ101) and small parts were measured and photographed with the camera attachment (Mshot-MSX2). The specimens kept in SYS and specimen photos from A, CAS, ISC, K, L, P and US were examined. Herbarium acronyms follow Thiers (2024). Terminology follows McClure (1940), Li et al. (2006) and Beentje (2016).

## ﻿Taxonomic treatment

### ﻿ Bambusa rushunii

Taxon classificationPlantaePoalesPoaceae

J.B.Ni & Y.H.Tong sp. nov.

B8AEC5AD-D2F3-5BAF-8229-F2F027C66E5F

urn:lsid:ipni.org:names:77357415-1

Figs 1 , 2

#### Type.

China. • Guangdong Province: Yangjiang City, Yangchun County, Tanshui Town; 22°4'52.71"N, 111°36'6.80"E; alt. 28 m; 18 September 2023; *Jing-Bo Ni et al. NJB-004* (holotype: IBSC!).

#### Diagnosis.

*Bambusarushunii* resembles *B.gibba* McClure and *B.dissimulator* McClure, but can be easily distinguished from *B.gibba* by having a thicker culm wall (ca. 1.5 cm vs. 3–5 mm), extremely shortened basal internode present (vs. absent), culm leaf sheath without (vs. with) a protuberance on higher shoulder and dark brown strigose on the central part (vs. glabrous wholly), higher culm leaf ligule (4–8 mm vs. 2–3 mm) and glabrous (vs. abaxially densely pubescent) foliage leaf and is different from *B.dissimulator* by the extremely shortened basal internode present (vs. absent), nearly truncate or slightly obliquely truncate (vs. asymmetrically convex) culm leaf sheath apex, culm leaf auricle contiguous (vs. not contiguous) with the blade base, culm leaf blade base not narrowed (vs. narrowed), foliage leaf ligule margin ciliate (vs. glabrous) and glabrous (vs. abaxially pubescent) foliage leaf.

**Figure 1.**
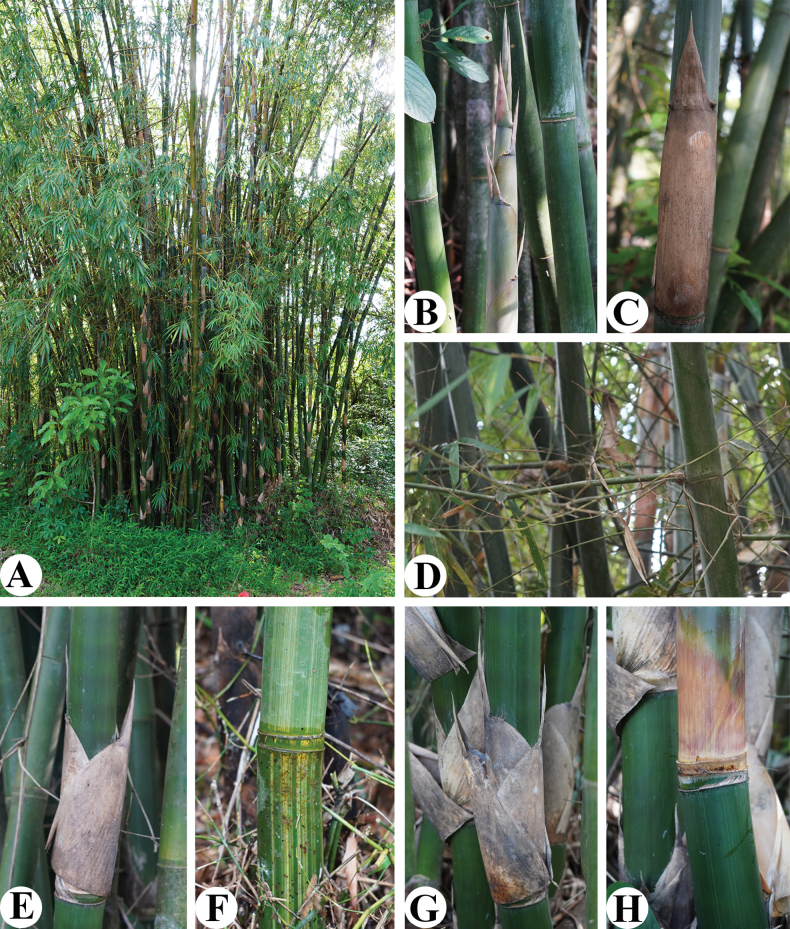
*Bambusarushunii* J. B. Ni & Y. H. Tong **A** habit **B** new shoot **C** culm leaf **D** branch complement **E–H** extremely shortened internodes. Photos by Meng-Ling Li.

#### Description.

Arborescent bamboo. Rhizome pachymorph, short-necked. Culm 8–10 m tall, 5–7.5 cm in diameter, erect, basal part slightly zigzag, apex slightly pendulous; internode terete, 35–45 cm long, 2^nd^ and/or 3^rd^ and/or 4^th^ internodes usually extremely shortened, only ca. 1 cm long; initially white powdery, glabrous, basal internodes green with many pale yellow stripes, stripes sometimes not inconspicuous; wall ca. 1.5 cm thick; supranodal ridge inconspicuous, sheath scars raised; culm bud round, branches developing from 5^th^ node upwards, nearly horizontally spreading, branch complements with several branches per node, central 3 dominant, branchlets usually specialised into weak thorns. Culm leaf sheath deciduous, thickly leathery, 30–40 cm long, 9–12 cm wide at apex, 20–32 cm wide at base, with yellow-green stripes, stripes inconspicuous when dry, sparsely dark brown strigose on central part, longitudinal ribs conspicuous when dry, apex nearly truncate or slightly obliquely truncate, without protuberance on shoulder; auricles unequal, oblong to lanceolate, contiguous with the base of blade, slightly wrinkled, margin densely with ca. 8 mm long and curved oral setae; larger auricle 1–2 cm long, 4–6 mm wide, not slanted; smaller auricle 3–8 mm long, 2–5 mm wide; ligule 4–8 mm high, margin serrate, densely with 2–3 mm long cilia; blade erect, narrowly triangular, 10–13 cm long, 5–8 cm wide, 1/2–3/5 as long as culm sheath, glabrous on both sides, apex involute and acuminate, base slightly extending outwards and joined with auricles, 3/5–4/5 as wide as sheath apex. Foliage leaves 7–11 per ultimate branchlet, sheath ca. 6 cm long, glabrous; auricles elliptic, ca. 2 mm long, ca. 1 mm wide, oral setae deciduous, ca. 8 mm long; inner ligules ca. 1 mm high, entire, margin ciliate; pseudopetioles ca. 2 mm long, ca. 1 mm wide; blades linear-lanceolate, papyraceous, 15–20 cm long, 1.4–1.7 cm wide, both surfaces glabrous, apex acuminate, base subrounded to cuneate, secondary veins 6 pairs, transverse veins inconspicuous. Pseudospikelets sessile, usually several to many fasciculate at nodes of flowering branches, green when fresh, yellow when dry, linear-lanceolate, 4–6 cm long, 5–8 mm wide, basally subtended by several gemmiferous bracts; prophylls ovate, 3–4 mm long, 2–keeled, apex densely ciliolate; gemmiferous bracts 1–3, ovate, 4–9 mm long, glabrous, 10–14-veined, apex acuminate and muronate; florets several to many, apical 1–2 sterile, rachilla segments flat, ca. 4 mm long, puberulous, slightly grooved, apex enlarged, with a ring of white hairs, disarticulating below each floret; glumes 1 or 2, broadly ovate, 5–6 mm long, glabrous, 10–12-veined, apex acute; lemma lanceolate, ca. 13 mm long, glabrous, 10–12-veined, apex acute; palea lanceolate, ca. 12 mm long, slightly shorter than lemma, apex acute, abaxially 2-keeled, keels densely ciliolate at apex, with 6 veins between keels and 4 veins on each side; lodicules 3, subequal, fleshy, white, ca. 2 mm long, margin with long cilia; stamens 6, filaments free, ca. 2 cm long, white; anthers initially green-yellow, later yellow-brown, ca. 5 mm long, apex retuse; ovary obvoid, ca. 1 mm long, apex hispidulous, style short, ca. 0.3 mm long, sparsely hispidulous at base; stigmas 3, 2–3 mm long, slender and plumose. Mature caryopsis fusiform, yellow, ca. 8 mm long.

**Figure 2.**
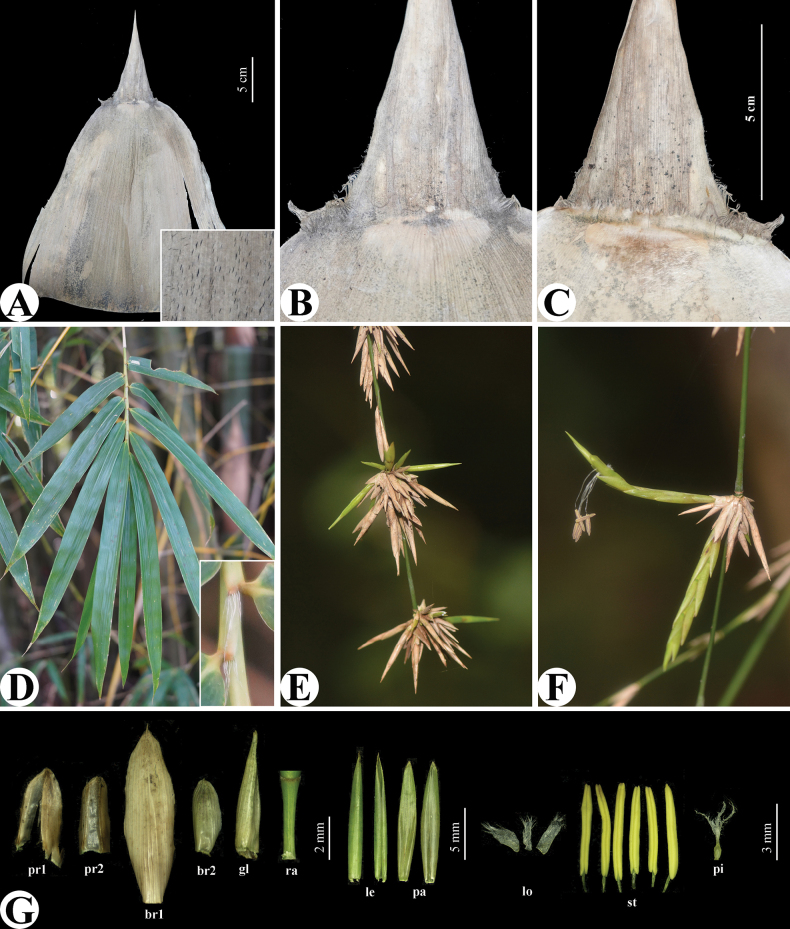
*Bambusarushunii* J. B. Ni & Y. H. Tong **A** abaxial view of culm leaf, with a close-up view of the strigose hairs on the central part **B** abaxial view of culm leaf sheath apex, showing auricles **C** adaxial view of culm leaf sheath apex, showing ligule **D** foliage leafy branchlet **E** flowering branches **F** pseudospikelets **G** dissection of pseudospikelet, pr1 and pr2 = prophylls, br1 and br2 = gemmiferous bracts, gl = glume, ra = rachilla segment, le = lemma, pa = palea, lo = lodicules, st = stamens, pi = pistil. Photos **A–D, G** by Meng-Ling Li; **E, F** by Shu-Peng Dong.

#### Phenology.

New culm shoots produced from July to September, flowering in September.

#### Distribution and habitat.

This new species is found in Yangjiang City, Guangdong Province, China and cultivated in South China Botanical Garden. It usually occurs near villages and streams at elevations of 20–120 m.

#### Etymology.

The species epithet honours Mr. Ru-Shun Lin, a retired employee from South China Botanical Garden, Chinese Academy of Sciences, who has made more than 600 living collections of bamboos and contributed a lot to the development of the Bamboo Garden of South China Botanical Garden. Its Chinese name is given as 汝顺坭簕竹(Pinyin: rǔ shùn ní lè zhú).

#### Discussion.

In "Flora of China", the genus *Bambusa* was further classified into four subgenera: subg. Bambusa, subg. Dendrocalamopsis L. C. Chia & H. L. Fung, subg. Leleba (Rumph. ex Nakai) Keng ex L. C. Chia & H. L. Feng and subg. Lingnania (McClure) L. C. Chia & H. L. Fung (Xia et al. 2006). *Bambusarushunii* possesses typical characteristics of subg. Bambusa, such as the relatively thick culm wall, persistent culm leaf blade with a broad base that is 1/2–3/4 as wide as sheath apex and the branchlets usually specialised into weak thorns. Thus, it is placed into that subgenus.

Amongst the species of B.subg.Bambusa, *B.rushunii* is most similar to *B.gibba* and *B.dissimulator* in the branchlets of lower branches which are usually specialised into weak thorns and the relatively small culm leaf auricles. The detailed morphological comparison of the three species is shown in Table 1 and the diagnosis section. The most unique morphological character of *B.rushunii* is the extremely shortened internodes at the culm base. As far as we know, this characteristic also occurs to another species of Bambusasubg.Dendrocalamposis, viz. *B.bicicatricata* (W. T. Lin) L. C. Chia & H. L. Fung. However, except this character, *B.rushunii* is very different from *B.bicicatricata* in many other characters, such as morphology of culm leaves, pseudospikelet length and number of stigmas, since they belong to different subgenera (Chia et al. 1996; Xia et al. 2006).

**Table 1.** Morphological comparisons of *Bambusarushunii*, *B.gibba* and *B.dissimulator*.

Characters	*B.rushunii*	*B.gibba*	*B.dissimulator*
Culm wall thickness	ca. 1.5 cm	3–5 mm	ca. 1.5 cm
Internode			
extremely shortened basal internodes	Present	Absent	Absent
indumentum	Glabrous	Sparsely stiffly grey-white or brown strigose, glabrescent	Glabrous or hairy
Culm leaf			
sheath indumentum	Dark brown strigose on the central part	Glabrous	Subglabrous or inconspicuously strigose
sheath apex	Truncate or slightly obliquely truncate	Obliquely truncate	Asymmetrically convex
protuberance on higher shoulder	Absent	Present	Absent
auricle	Contiguous with the base of the blade	Contiguous with the base of the blade	Not contiguous with the blade
ligule height	4–8 mm	2–3 mm	5–7 mm
blade	Base not narrowed 3/5–4/5 as wide as sheath apex	Base not narrowed ca. 2/3 as wide as sheath apex	Base cordately narrowed 1/2–3/5 as wide as sheath apex
Foliage leaf ligule margin	Ciliate	Ciliate or glabrous	Glabrous
Foliage leaf indumentum	Glabrous	Abaxially densely pubescent	Abaxially sparsely pubescent

#### Additional specimens examined.

*Bambusarushunii* J. B. Ni & Y. H. Tong: China. • Guangdong Province, Guangzhou City, Tianhe District, introduced from the type locality, cultivated in Bamboo Garden of South China National Botanical Garden, 5 September 2024, *J. B. Ni 005* (paratype: IBSC).

*Bambusadissimulator* McClure: China. • Guangdong Province, Guangzhou City, Panyu District [Haizhu District], Lingnan University campus (now the campus of Sun Yat-Sen University), 26 September 1939, *F. A. McClure 20861* (K000854766, image); • ibid., 30 April 1931, *H. Fung A-674*/*BG2348* (A00023169, image, L0043812, image, SYS00095355, US00130308, image, US00130310, image, US00130311, image, US00130312, image); • ibid., 18 November 1929, *H. Fung LU18499* (SYS00011949, US00391111, image); • ibid., 30 April 1931, *H. Fung LU19079* (isotypes: CAS0027955, image, ISC-v-0000942, image, ISC-v-0000943, image, K000854765, image, L0043812, image); • ibid., 15 March 1932, *H. Fung 20003* (two sheets: SYS00011892 & SYS00011893); • ibid., Lingnan University, Primary School, 30 October 1936, *H. Fung 20987* (US0050544, image).

Bambusadissimulatorvar.albonodia McClure: China. • Guangdong Province, Guangzhou City, Honam Island [Haizhu District], west end of same island of land, Lingnan University Agriculture workmen’s barracks, 13 December 1937, *F. A. McClure 20719* (holotype: two sheets US00130313 & US0034812, image); • ibid., Honam Island [Haizhu District], Ng Ts’uen, 18 May 1921, *F. A. McClure LU18552* (two sheets: US 00034813 & US0034814, image).

Bambusadissimulatorvar.hispida McClure: China. • Guangdong Province, Guangzhou City, Panyu District [Haizhu District], growing on edge of small knoll, east. of Lingnan University campus (now the campus of Sun Yat-Sen University), 26 September 1939, *F. A. McClure 20861* (holotype US00130315, image; isotypes A00023170, image, ISC-v-0000944, image, K00854764, image, K00854766, image, L0043813, image, P00800933, image).

*Bambusagibba* McClure: China. • Kiangsi [Guangxi], south of Kanchow [Qinzhou], cultivated in Lingnan University Bamboo Garden (now in the campus of Sun Yat-Sen University), 30 September 1933, *H. Fung 20709* (holotype: three sheets US00065370, US00065371 & US00065372, image; isotype: three sheets US00289540, US00289541 & US00289542, image); • ibid., March 1929, *F. A. McClure LU18518* (A00023177, image, K000854759, image, L0043815, image, L0043816, image, P00800942, image SYS00095349); • ibid., 23 February 1937, *H. Fung 21001* (SYS00095350, SYS00095351).

## Supplementary Material

XML Treatment for Bambusa rushunii

## ﻿Additional information

### Author ORCIDs

Meng-Ling Li https://orcid.org/0009-0006-0264-4485

Shu-Peng Dong https://orcid.org/0009-0004-0409-9551

Yi-Hua Tong https://orcid.org/0000-0002-5034-005X

### Data availability

All of the data that support the findings of this study are available in the main text.
